# Compulsive Sexual Behavior and Alcohol Use Disorder Treated With Naltrexone: A Case Report and Literature Review

**DOI:** 10.7759/cureus.25804

**Published:** 2022-06-09

**Authors:** Tania Sultana, Javeria Sahib Din

**Affiliations:** 1 Psychopharmacology Research Program, The Manhattan Psychiatric Center, New York City, USA; 2 Psychiatry, Bergen New Bridge Medical Center, Paramus, USA

**Keywords:** psychotherapy, alcohol use disorder, dopamine pathway, naltrexone, compulsive sexual behavior or sexual addiction

## Abstract

Compulsive sexual behavior (CSB) or sexual addiction is a term that generally indicates excessive and uncontrolled sexual behavior. This may lead to subjective distress, social and occupational impairment, or legal and financial consequences. Often, this condition is underreported and untreated. Until now there are no FDA-approved medications for sexual addiction or compulsive sexual behaviors. However, the therapeutic benefits of selective serotonin reuptake inhibitors (SSRIs) and naltrexone are known. This is a case of a 53-year-old male with a history of extensive alcohol use, alcohol withdrawal seizure, and delirium tremens. The patient was treated with naltrexone 50 mg/day for alcohol use disorder. The patient reported that his “sexual compulsion” also reduced after the medication and there was an improvement in both alcohol addiction and self-reported compulsive sexual behavior. This case report also includes a literature review of pharmacotherapy, especially naltrexone, for the treatment of sexual addiction/compulsive sexual behavior. The literature review has shown that patients’ symptoms were improved in different doses without side effects, and based on this and our experience, it can be said that naltrexone is effective in the reduction and remission of the symptoms of CSB or sexual addiction.

## Introduction

Based on clinical and epidemiological evidence, hypersexual behavior and disorder are described as non-paraphilic excesses of sexual desire and activity with an impulsivity component and accompanied by clinically significant personal distress, and social and medical morbidity. The estimated prevalence rate in the general population is 3-6%. Problematic behaviors include excessive masturbation, cybersex, pornography sex, sexual behavior with consenting adults, telephone sex, strip club visitation, and others [1,2]. Previously, in 1991, Coleman et al. described compulsive sexual behavior (CSB) as involving a wide range of paraphilic and non-paraphilic symptoms. Paraphilic CSB involves unconventional sexual behaviors in which there is a disturbance in the object of sexual gratification or the expression of the sexual gratifications. On the other hand, non-paraphilic CSB involves conventional sexual behavior that has become excessive or uncontrolled [3]. Because of the highly negative consequences of these behaviors in the personal, family, and social life; appropriate screening tools, assessment, and diagnosis as well as the development of an appropriate model for the treatment of sexual addiction or CSB has paramount importance.

The etiology of sexual addiction is multifactorial and still unknown; Rosenberg et al. proposed increased dopamine levels as the underlying contributing factor for compulsive sexual behavior [4]. Other possible causal or contributing factors related to hypersexual behavior include epigenetic changes, dysregulated hypothalamo-pituitary-adrenal axis, sexual abuse, or other traumatic experiences such as psychological abuse. CSB can also be a manifestation of other disorders mainly neuropsychiatric and psychiatric disorders [5]. Clinicians in this field recommend multifaceted treatment approaches including various types of psychotherapy and psychopharmacological treatment. Several pharmacological interventions (eg. naltrexone, selective serotonin reuptake inhibitors (SSRIs), citalopram, clomipramine, nefazodone, leuprolide acetate, valproic acid) have been used and reported in several case reports [6]. Naltrexone is an opiate antagonist approved initially for opiate use disorder (in the 1960s) and later for the treatment of alcohol use disorder (in 1994) [7]. Recently, off-label use of naltrexone has been shown to reduce the symptoms of sexual addiction, hypersexual behavior, or CSB and disorder, as evident in several case reports, case series, and open-label trials [8,9,10,11,12]. This case report includes a detailed literature review related to sexual addiction or CSB and treatment strategies. The authors also investigate the therapeutic response or outcome of naltrexone on sexual addiction or CSB based on available evidence in the literature.

## Case presentation

We present the case of a 53-year-old male with an extensive history of alcohol use, alcohol withdrawal seizures, and delirium tremens, who has undergone psychosocial stressors including the passing of his father about a month ago, job insecurity, and poor social support, presented with depression and suicidal ideation in the context of alcohol intoxication. The patient reported “heavy” drinking daily including an “eye-opener” in the morning. During the evaluation, the patient was actively withdrawing from alcohol with an elevated Clinical Institute Withdrawal Assessment (CIWA) score of 16. His blood alcohol level was 330. The patient also reported insomnia, poor appetite, and excessive worry but denied current anhedonia, loss of energy, poor concentration, and feeling of hopelessness. The patient denied current suicidal/homicidal ideation/intent/plan. Symptoms of psychosis and mania were not reported or observed. 

The patient had a history of hospitalization due to an alcohol withdrawal seizure and an episode of delirium tremons last year. There was no history of prior psychiatric hospitalization, medication trial, and outpatient treatment. The patient reported a history of depressive symptoms of sad mood, poor energy and concentration, and anhedonia. The patient also reported a history of anxiety symptoms of excessive worry and fatigue. He denied the use of illicit drugs.

The patient was started on antidepressant sertraline and naltrexone 50mg daily to address depression and alcohol use disorder. Surprisingly, the patient reported that he had had unusual sexual urges for about two years that were difficult to control. His CSB was characterized by excessive use of pornography and compulsive masturbation resulting in some degree of functional impairment in his daily and social life. After a month of starting naltrexone 50 mg daily, he observed that he significantly decreased using pornography and compulsive masturbation. This also improved his daily functioning. The patient was continued on the treatment and reported persistent improvement in sexual urges or CSB.

## Discussion

Formalized criteria for diagnosed CSB are not established yet, mainly due to a lack of research as well as the heterogeneous presentation of the condition. Some patients present with clinical features that resemble an addictive disorder, some demonstrate elements of impulse control disorder, and others act out in a way that resembles obsessive-compulsive disorder [7]. Besides, CSB presents as a symptom of many psychiatric disorders (e.g., manic episodes, depressive disorder, substance use disorder, borderline personality disorder) and neuropsychiatric disorders (e.g., frontal and temporal lobe lesion, dementia), and is related to using certain medication (e.g., L-dopa for the treatment of Parkinson) and illicit drugs such as methamphetamine. Oftentimes, CSB related to these conditions does not fulfill the criteria of compulsive sexual behavior disorder (CSBD) described in ICD-11 for mortality and morbidity (version 04/2019).

ICD-11 diagnostic guidelines for CSBD [11,5].

“Compulsive sexual behavior disorder is characterized by a pattern of failure to control intense, repetitive sexual impulses or urges resulting in repetitive sexual behavior. Symptoms may include repetitive sexual activities becoming a central focus of the person’s life to the point of neglecting health and personal care or other interests, activities, and responsibilities; numerous unsuccessful efforts to significantly reduce repetitive sexual behavior, and continued repetitive sexual behavior despite adverse consequences or deriving little or no satisfaction from it. The pattern of failure to control intense, sexual impulses or urges and resulting repetitive sexual behavior is manifested over an extended period of time (e.g., 6 months or more), and causes marked distress or significant impairment in personal, family, social, educational, occupational, or other important areas of functioning. Distress that is entirely related to moral judgments and disapproval about sexual impulses, urges, or behaviors is not sufficient to meet this requirement”

Also, if CSB is a symptom of such disorders, CSBD diagnosis should not be considered [5]. In addition, identifying a CSBD is a challenge because of its sensitive and personal nature. Unless the patient presents for the treatment of this condition, they are reluctant to discuss it [13]. In this presenting case, the CSB was related to alcohol use disorder (AUD) and did not fulfill the criteria of CSBD.

There has been growing research on the evidence of biological, psychological, and social contributing factors to this condition. The neurobiology of pleasurable responses from various behaviors, experiences, or artificial substances is explained by many scholars mostly involving the activation of the dopaminergic pathways by stimulation of opiate receptors. Natural or artificial stimulation of opiate receptors increases dopamine levels via decreasing inhibition of dopamine pathways, which creates a feeling of pleasure [14]. Continuous activation of dopamine pathways leads to the downregulation of dopamine thought to result in craving seen in addictive disorders [7]. Abnormal dopamine levels have been proposed as an underlying cause or contributing factor to excessive sexual behavior [4]. Dopamine plays an important role in neurobiology, some of the functions of dopamine include movement, memory, pleasure, behavior, cognition, mood, sleep, sexual arousal, and prolactin regulation [7]. Also, some studies have suggested the interaction between negative reinforcement (anxiety reduction) and positive reinforcement (gratification through excitation and orgasm), which might be related to imbalances in different neurotransmitters such as dopaminergic and serotonergic systems [5].

Jokinen et al 2017 showed that epigenetic changes in the corticotropin-releasing hormone gene region were related to hypersexual behavior [15]. A separate study showed the hypothalamo-pituitary-adrenal axis was dysregulated in men with hypersexual disorder. This dysregulation might correspond to sexual abuse or traumatic experiences such as psychological abuse [5]. Psychological correlates in CSB are attachment problems and can be associated with traumatic experiences [16]. In some individuals, sexuality is used as a strategy to self-medicate and cope with negative emotions such as depression [17]. Negative attitudes towards sexuality and pornography consumption are related to social factors. Digital media and associated availability of pornography, as well as factors such as religiousness and moral disapproval of pornography use also influence the development of CSBD at a societal level [5].

Screening tools or measurements to identify someone at risk of developing CSB were developed by Patrick Carles in 1991. This Sexual Addiction Screening Test is a 25-item, self-reported symptoms checklist. Screening tests could identify at-risk behavior that requires further clinical exploration [18]. Later, Kafka suggested a behavioral screening test (i.e. Total Sexual Outlet) in which seven sexual orgasms per week regardless of how they are achieved, could be at risk of developing CSB and require further clinical exploration [13]. Several developments have been made regarding measuring the instrument of CSB and CSBD. The most researched self-rating measurements of hypersexual disorders are the Hypersexual Screening Inventory, the Hypersexual Behavior Inventory (HBI-19), the Sexual Compulsivity Scale, the Sexual Addiction Screening Test, the Sexual Addiction Screening Test-Revised, and the Compulsive Sexual Behavior Inventory. One of the self-rating scales is combined with an external rating of ICD-11 criteria for a thorough evaluation [5,19,20,21]. 

Each patient with CSB should have an individualized and multimodal therapeutic approach that includes specific psychotherapy as well as pharmacotherapy [5]. Individualized psychotherapy varies but the most common approaches are cognitive behavioral therapy (CBT) and psychodynamic psychotherapy. CBT in CSBs focuses on identifying triggers and reshaping the cognitive distortion of sexual behaviors and emphasizes relapse prevention. Psychodynamic psychotherapy in CSB explores the core conflicts that drive dysfunctional sexual behavior. Family therapy and couple therapy are also helpful [13]. Therapeutic approaches for CSBD can be based on different models such as the Dual-Control Model, and the Sexual Tipping Point Model. These integrated models of CSBD are aimed at bringing a more flexible balance between sexual inhibition and excitation. This balance can be achieved by improving sexual self-control. Psychotherapy for CSBD includes CBT and acceptance and commitment therapy (ACT), and pharmacotherapy includes SSRIs such as escitalopram and paroxetine, naltrexone, and testosterone lowering agents [5]. 

Based on published literature on naltrexone use (off-label) for the treatment of CSB, CSBD, and sexual addiction induced by dopamine replacement therapy, complete control over sexual urges is achieved in the dose range of 100-150mg/day. Naltrexone is used after establishing normal liver and kidney function tests. Grant et al. (2001) published a case report of a 58-year-old male with kleptomania and CSB who failed to respond to fluoxetine, behavioral therapy, and psychotherapy, and achieved remission on high doses of naltrexone (150mg/day). Discontinuation and rechallenge further supported their result [10]. Raymond et al. (2002) reported a case series of two cases, a 42-year-old woman with major depressive disorder and CSB, symptoms of anxiety, and depression were improved by fluoxetine 60mg/day but did not reduce the symptoms of CSB. Naltrexone 50mg/day decreased the symptoms of CSB initially and she had remission from the sexual urge and was urged to use cocaine on naltrexone 100mg/day. In the second case, a 62-year-old male with a 20-year history of intermittent CSB and failed trials of fluoxetine, citalopram, bupropion, and buspirone was successfully treated with naltrexone 100mg/day [8]. Rayback et al. (2004) studied naltrexone’s efficacy on adolescent sex offenders. Most participants reported decreased arousal, masturbation, sexual fantasies, and increased control over sexual urges between the doses of 100-200 mg/kg [22]. Bostwick et al. (2008) reported a case of a 24-year-old male who presented with an internet sex addiction and developed complete control over his impulses when the naltrexone dose was titrated up to 150mg/day. Later, the patient gradually lowered the dose and was stable on naltrexone 50mg/day. He was on SSRI and had also tried group and individual psychotherapy, Sexual Addicts Anonymous, and pastoral counseling with no improvement [12]. Camacho et al. (2018) reported a case of a 27-year-old male with self-reported “sexual compulsion” that did not improve while on fluoxetine 40mg/day and aripiprazole 10mg/day, who reported significant improvement on naltrexone 50-100mg/day [23]. 

Verholleman et al. (2020) presented a case in the systematic review on naltrexone treatment for hypersexuality induced by dopamine replacement therapy. A 65-year-old Caucasian male had developed sexual addiction while he was on treatment for Perkinson disease. This was effectively treated with naltrexone 50mg/day [18]. Savard et al. (2020) published a prospective pilot study on 20 male patients (mean age=38.8) with a diagnosis of CSBD treated with naltrexone 50mg/day for four weeks. Their result suggests that naltrexone is feasible, tolerable, and may reduce the symptoms of CSBD. This study provides novel insight into the pharmacological intervention of CSBD [24].

## Conclusions

From the case in this report, it can be seen that naltrexone is effective for sexual addiction and CSD at various doses. However, it is important to establish the efficacy and tolerability through randomized controlled trials because this behavior is not uncommon and has psychiatric and medical consequences. 
